# Prevalence of HIV and willingness to uptake preventive services among female sex workers in Kano State, Nigeria

**DOI:** 10.1371/journal.pone.0319942

**Published:** 2025-04-10

**Authors:** Godwin Omokhagbo Emmanuel, Paul Umoh, Paul Amechi, Olaniyi Felix Sanni, Roger Abang, Ochonye Bartholomew Boniface, Ismaeel Mohammed Yahaya, Agie Muhmmad Auwal

**Affiliations:** 1 Heartland Alliance LTD/GTE, Abuja Nigeria,; 2 Litsam Youth Awareness Initiative, Kano, Nigeria; HIV/STI Surveillance Research Center and WHO Collaborating Center for HIV Surveillance, Institute for Future Studies in Health, Kerman University of Medical Sciences, IRAN, REPUBLIC OF ISLAMIC

## Abstract

**Background:**

HIV remains a critical global health issue, significantly affecting key populations, particularly female sex workers who face socio-economic hardships amidst limited healthcare access and high partner turnover. This study aimed to determine the HIV prevalence and to understand FSWs’ willingness to use HIV services in Kano State, Nigeria, to inform effective intervention strategies.

**Method:**

This cross-sectional survey was conducted in May 2023 among FSWs in three Local Government Areas of Kano State (Gwale, Fagge, and Tarauni), Nigeria. The participants were recruited from nightclubs and brothels across the selected communities using the snowball sampling technique. The analysis of data included descriptive statistics and logistics binary regression. Using a structured questionnaire, data were collected from 650 FSWs, all of whom were tested for HIV. Participants scoring 75% or higher on willingness to use HIV services were deemed willing. Data were analysed using Microsoft Excel and SPSS Version 28.0.

**Results:**

The study involved 650 FSWs across three LGAs in Kano State, with an HIV prevalence of 8.5% and 65.8% reporting willingness to access HIV services. Willingness to take HIV Self-Testing (96.0%) and Pre-exposure Prophylaxis (66.8%) services was high, primarily driven by the desire to know and confirm HIV status (87.5%). Barriers included stigma and harassment (35.4%) and lack of free, accessible services (29.2%). FSWs in Fagge and Gwale were significantly less willing to access services compared to those in Tarauni (AOR: 0.068 and 0.180, P < 0.05). Those with a sexual debut at 18 years or above were less willing (COR: 0.569, P = 0.013), while having more than one sexual partner (COR: 1.574, P = 0.038), recent vaginal sex (AOR: 5.182, P = 0.039), and engagement in transactional sex (e.g., paid for sex, COR: 2.304, P = 0.005) were associated with higher willingness.

**Conclusion:**

This study highlights the role of socio-economic challenges and stigma in shaping HIV prevalence and service uptake among female sex workers in Kano. Holistic interventions and innovative approaches are essential to address barriers, improve accessibility, and ensure sustainable outcomes.

## Introduction

Human Immunodeficiency Virus (HIV) continues to pose a substantial public health challenge on a global scale, with an estimated 39.9 million people living with HIV as of 2023.[1] During the same year, 1.3 million individuals became newly infected, 630,000 people succumbed to AIDS-related illnesses, and 30.7 million were receiving antiretroviral therapy.[1] HIV continues to disproportionately affect specific populations, particularly female sex workers (FSWs), despite recent advancements in treatment and prevention. HIV prevalence rates among FSWs are significantly higher than those of the general population on a global scale, with some regions reporting prevalence rates exceeding 30%.[2,3] Factors such as socio-economic vulnerabilities, limited access to healthcare services, and high rates of partner turnover increase the risk of HIV for FSWs. [2,3]

The situation is particularly severe in Sub-Saharan Africa, which is the epicentre of the HIV epidemic. The region is the site of nearly 70% of the global HIV burden, with millions of new infections and fatalities occurring annually.[4] FSWs are a critical category for intervention in this context, as their risk is significantly elevated. Studies have demonstrated that the prevalence of HIV among FSWs in Sub-Saharan Africa can reach as high as 50%, contingent upon the country and specific context.[5] Nigeria is responsible for a significant portion of the regional burden, with an estimated 1.9 million individuals living with HIV as of 2021, according to a Statista Research Department publication in February 2023.[6,7] The National Agency for the Control of AIDS (NACA) estimated 13.0% HIV prevalence among FSWs based on the stigma index survey of people living with HIV in Nigeria as of 2021.[8–11] The imperative necessity for targeted interventions is underscored by the stark contrast between the national prevalence rate of 1.4% and this figure. In 2023, the Kano State government tested 138,430 individuals for HIV, identified 4,728 as positive, and successfully initiated 4,140 of them on antiretroviral therapy (ART) [12].

However, in the battle against the epidemic, the willingness of FSWs in Nigeria to access HIV services is a critical factor. Barriers such as fear of stigma, fear of side effects, discrimination, transportation, distance, and financial challenges substantially impedes the adoption of HIV services [13–15]. Despite progress in HIV testing and treatment, limited research exists on the barriers FSWs face in accessing services in Kano state. Understanding these challenges is crucial for developing targeted interventions to improve service uptake and reduce HIV prevalence in this vulnerable population. This study aims to assess the barriers and facilitators influencing the uptake of HIV services among FSWs in Nigeria, with the goal of informing targeted interventions to improve service accessibility and reduce HIV prevalence in this population.

## Methodology

### Study design

This was a cross-sectional study involving data collection from FSWs in the night clubs and brothels across three Northern Local Government Areas (LGAs) of Kano, Nigeria, in November 2023. Participants were tested for HIV using blood-based self-test kits, requiring a fingerstick, with results available in 15 minutes. Positive cases were linked to treatment, and willingness to access HIV services was assessed using 17 well-structured questions. The threshold for willingness was set at 75%. The 17-point questions include willingness to attend regular meetings organised to discuss HIV-related issues, allow people to ask you questions about your risk behaviour, follow a plan to help address your HIV risk, receive and use condoms and lubricants, visit clinics for STI check-ups and cervical cancer screening, go to public, private, or peer-led clinics to get HIV-related services, attend public, private, or peer-led clinics if accompanied by peers, have peers facilitate access to services in public or private hospitals when encounter difficulties, have peers assist in getting voluntary HTS, and have peers serve as drug adherence supporters if HIV positive. Responses were set as “unwilling” and “willing”, with 0 and 1 scores, respectively.

### Study area and study population

This study was conducted in three LGAs of Kano State: Gwale, Fagge, and Tarauni, which are part of the rapidly urbanizing Kano metropolis, the commercial hub of Northern Nigeria. As of 2020, population densities were approximately 18,937 people per square kilometer in Gwale, 9,180 in Fagge, and 15,722 in Tarauni [16]. These areas exhibit higher economic activity compared to rural regions, driving increased demand for healthcare services, including HIV-related care. However, healthcare facility distribution is uneven, with some areas more served than others [17]. The participants were recruited from nightclubs and brothels across the selected communities using the snowball sampling technique. The recruitment process began with initial participants, known as seeds, who were identified through key informants familiar with the FSW networks in these locations. These seeds referred other participants, facilitating access to a population that might otherwise be difficult to reach. Recruitment was conducted discreetly to ensure participants’ privacy and comfort, with informed consent obtained before participation. Gaining the trust of participants and ensuring confidentiality in a stigmatized environment was the challenge faced during recruitment process. These challenges were mitigated by working with trusted community members and sensitizing participants about the study's purpose, the voluntary nature of participation, and the measures taken to protect their identities.

### Eligibility criteria

The study includes FSWs who, in this context, are women who exchange sexual services for money, goods, or other forms of compensation. This definition encompasses both those who engage in full-time sex work and those who participate part-time or occasionally. FSWs eligible for the study were aged 18 and above and resided in the selected LGAs of Kano State (Gwale, Fagge, and Tarauni). Only FSWs who could communicate in English, Nigerian Pidgin, Hausa, or other local dialects commonly spoken in the study areas were included, this was done to ensure transparency. Additionally, participants who did not grant consent or were under 18 years of age were excluded from the study.

### Sample size determination

The total population sizes for the selected LGAs were obtained from census estimates and were as follows: Fagge (1,282,500), Gwale (588,500) and Tarauni (364,900). The sample size of the three selected LGA in Kano State was obtained from the 2022 population projection of Kano State.[19] The sample size for this study was calculated using a 5.8% prevalence of HIV among key populations in Kano State, according to Adeoye *et al*.[18] and single proportion formula:


$$n\;=\;\frac{Z^{2}\;\times \;p\;\times \;(1\;-\;p)\;}{E^{2}}$$

Where Z is the confidence level with Z-value for a 95% confidence level =  1.96

Prevalence of HIV among KPs in Kano State p = 5.8%, = 0.058

E is the margin of error, often set at 2% or 0.02

Adding 20% attrition, n = 525 + 105, = 630

The minimum sample size for each Local Government Area (LGA) was influenced by the accessibility of participants and their willingness to engage during data collection. While proportional allocation based on population sizes initially guided the sampling plan, practical considerations such as recruitment feasibility and participant availability resulted in slightly adjusted distributions.

### Sampling techniques

This study adopted a quantitative research approach for comprehensive data collection and analysis. A combination of snowball sampling and convenience sampling techniques was used. Initial participants were recruited from brothels and clubs across the three LGAs in Kano State based on their accessibility and willingness to participate (convenience sampling). These initial participants, known as “seeds,” referred others within their networks, expanding the sample through the snowball sampling method. This approach facilitated access to a hard-to-reach population while ensuring privacy and trust.

### Variables

The study's dependent variables were the prevalence of HIV among the participants and their willingness to take up HIV-related services. These variables aimed to capture both the health outcomes and service uptake behaviours of this high-risk population. This study examined a range of demographic, behavioural, and sexual health-related variables to understand factors associated with HIV prevalence and willingness to access HIV services among FSWs. The demographic variables included age, categorized into four groups: 18–24 years, 25–34 years, 35–45 years, and above 45 years. Educational level was also assessed, ranging from no formal education to tertiary education. Participants were further categorized by their Local Government Area (Fagge, Gwale, or Tarauni). Behavioural variables explored included drug abuse (whether participants had ever abused drugs) and drug injection (whether participants had ever injected drugs). Sexual behaviour was examined through the age of first sexual debut, classified as below 13 years, 14–17 years, or 18 years and above, as well as the reasons for first sexual intercourse, which included having fun or love, peer pressure, rape or forced intercourse, financial motives, or marriage. Participants’ recent sexual activity was assessed, including sexual activity in the last three months, the number of sexual partners during this period (one or more than one), and engagement in vaginal, anal, or oral sex within the same timeframe. The study also investigated transactional sex practices, distinguishing between those who did not engage in transactional sex, those who paid for sex, those who both paid for and received payment or gifts for sex, and those who only received gifts in exchange for sex. Condom use was another key variable, evaluated both for consistency of use in the last three months (always or irregularly) and use during the last sexual encounter (used or not used).

### Study instrument

The survey utilised a well-structured, open-ended and closed-ended questionnaire to collect primary qualitative data from the consented FSWs. The questionnaire was sectionalised into socio-demographics, willingness to take HIV services, HIV status, and reasons for and against taking HIV services. Self-test kits were provided for the participants to determine their HIV status. The structured questionnaire used in this study was pre-tested among a small sample of female sex workers outside the study area to ensure clarity, relevance, and reliability. Feedback from the pre-test informed necessary revisions, improving the questionnaire’s validity for the target population.

### Ethical consideration

The study protocol was reviewed and approved by the Health Research Ethics Committee of the Ministry of Health, Kano State, Nigeria, with approval number HNREC/17/03/2018. The FSWs consents were sought, and they were apprised of their prerogative to withdraw from the survey at any juncture without being obligated to furnish justifications for their choice. Confidentiality and anonymity were maintained by assigning unique codes instead of personal identifiers, and all data were securely stored with access restricted to the research team to ensure ethical compliance given the sensitivity of the study population.

### Data analysis

The data collected from FSWs were thoroughly cleaned by identifying and removing incomplete entries, duplicate responses, and any inconsistencies in the dataset. Outliers were carefully reviewed to ensure they did not skew the results, and invalid HIV self-test results were excluded from the analysis. The collated data were recorded, cleaned, and analysed using Microsoft Excel and Statistical Package for the Social Sciences (SPSS) for Windows Version 28.0. The analysis of data included descriptive statistics, frequency distribution, and logistics binary regression, and these statistics were represented in tables and figures to facilitate comprehensive interpretation. Binary logistics regression was used to analyse the influence of socio-demographic variables on willingness to take HIV services. Various reasons for and against HIV services updates were presented with charts.

## RESULTS

### Socio-demographic characteristics of the respondents

As shown in Table 1, most of the FSWs (46.3%, n = 37.2) were 25–34 years old, followed by 37.2% (n = 242) for 18–24 years. One of every five (20.6%, n = 134) of the population had no formal education, 49.2% (n = 320) attained secondary education, and 20.6% (n = 134) tertiary education. More than half abused substance (55.4%, n = 360) and 30.2% (n = 192) inject drugs. A significant proportion (27.2%, n = 177) of the FSWs had their first sexual experience before age 13 and 41.4% (n = 269) between age 14 and 17 years. Fun or affection (54.5%, n = 354), peer pressure financial need (26.5%, n = 172) were the major reasons for the first sexual debut among the respondents, while 2.6% (n = 17) were raped. The majority of the respondents (86.0%, n = 559) were sexually active in the past three months, with 68.5% (n = 445) having multiple partners. Oral sex prevalence was 10.0% (n = 65), while anal and vaginal were 4.9% (n = 32) and 84.2% (n = 547), respectively, while 38.0% (n = 247) received gifts for sex. In the past three months, condom use always was 39.1% (n = 254), and 52.9% (n = 344) used condoms during their most recent sexual encounter, while 32.9% (n = 214) did not.

**Table 1.** Socio-demographic Characteristics of the Respondents.

Variable	Parameter (n = 650)	Frequency	Percentage
Age	18–24	242	37.2
	25–34	301	46.3
	35–45	67	10.3
	Above 45	40	6.2
Education level	No formal education	134	20.6
	Primary	62	9.5
	Secondary	320	49.2
	Tertiary	134	20.6
Local Government Area	Fagge	200	30.8
	Gwale	250	38.5
	Tarauni	200	30.8
Drugs abuse	Never Abused Drugs	290	44.6
	Ever Abused Drugs	360	55.4
Drugs injection	Never Injected Drugs	454	69.8
	Ever Injected Drugs	196	30.2
Age of first sexual debut (years)	Below 13	177	27.2
	14 – 17	269	41.4
	18 and above	179	27.5
Reason for first sexual intercourse	Having fun/love	354	54.5
	To obtain money	172	26.5
	Peer pressure	62	9.5
	Rape/Forced	17	2.6
	Marriage	2	0.3
Sexually active in the last three months	Not Sexually Active	91	14.0
	Sexually Active	559	86.0
Sexual partners during the last three months	One	114	17.5
	More than one	445	68.5
Vaginal sex in the last three months	Not Have Vaginal Sex	12	1.8
	Have Vaginal Sex	547	84.2
Anal sex in the last three months	Not Have Anal Sex	527	81.1
	Have Anal Sex	32	4.9
Oral sex in the last three months	Not Have Oral Sex	494	76.0
	Have Oral Sex	65	10.0
Transactional sex engaged in the last three months	No transactional sex	67	10.3
	Paid & received	69	10.6
	Paid for sex	176	27.1
	Received gift for sex	247	38.0
Condom use in the last three months	Always	254	39.1
	Irregularly	305	46.9
Condom use during the last sexual encounter	Not Used Condom	214	32.9
	Used Condom	344	52.9

### Prevalence of HIV among FSWs in Kano state

**Fig 1:**
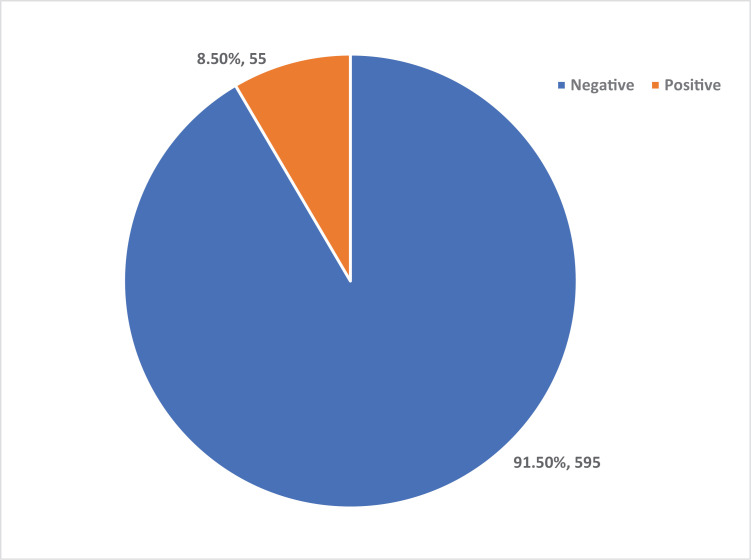
Prevalence of HIV among FSWs in Kano State.

As illustrated in Fig. 1, the HIV prevalence among FSWs in the three selected local government areas was 8.5% (n = 55), while 91.5% (n = 595) tested negative for HIV.

### Willingness to take HIV services

**Fig 2:**
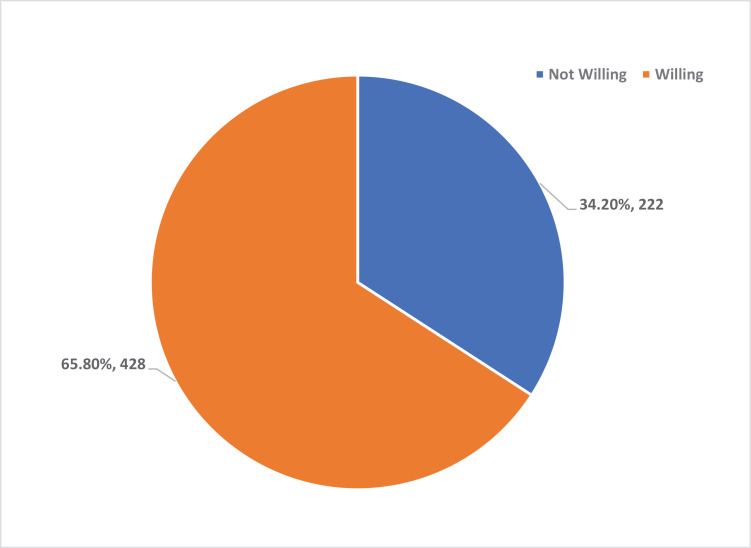
Willingness to Take HIV services among FSWs in kano state.

Fig. 2 illustrates that a significant proportion of FSWs in Kano State were willing to access HIV services (65.8%, n = 428), while 34.2% (n = 222) were unwilling.

### Factors associated with willingness to take HIV service among FSWs

Table 2 reveals significant factors affecting FSWs’ willingness to take up HIV preventive services. The age category shows no significant difference, although those aged 35–45 and above 45 exhibit a lower willingness with CORs of 0.620 and 0.839, respectively. Education level does not significantly influence willingness, though those with primary education show a higher willingness (COR: 1.459). Notably, the LGA is a significant factor; FSWs in Fagge and Gwale are significantly less willing to take up services with CORs of 0.097 and 0.228, respectively, and these remain significant in the adjusted model with AORs of 0.068 and 0.180 (P < 0.05). Drug abuse and drug injection status do not significantly impact willingness. However, the age of first sexual debut shows that those who had their debut at 18 years and above are significantly less willing (COR: 0.569, P = 0.013) compared to those below 13 years, though this is not significant in the adjusted model.

**Table 2.** Factors associated with willingness to take up HIV preventive services among FSWs.

Variable	Not Willing n (%)	Willing n (%)	COR (95% CI)	P-value	AOR (95% CI)	P-value
**Willing to take HIV services**	222 (34.2)	428 (65.8)	–	–	–	–
**Age category (Years)**						
18–24	81 (33.5)	161 (66.5)	**Ref**	–	–	–
25–34	96 (31.9)	205 (68.1)	1.074 [0.749–1.541]	0.697	1.006 [0.630–1.605]	0.981
35–45	30 (44.8)	37 (55.2)	0.620 [0.358–1.076]	0.089	0.804 [0.397–1.628]	0.544
Above 45	15 (37.5)	25 (62.5)	0.839 [0.419–1.678]	0.619	2.248 [0.841–6.006]	0.106
**Respondent Education Level**						
No formal education	40 (29.9)	94 (70.1)	**Ref**	–	–	–
Primary	14 (22.6)	48 (77.4)	1.459 [0.724–2.941]	0.291	1.361 [0.567–3.266]	0.490
Secondary	117 (36.6)	203 (63.4)	0.738 [0.478–1.140]	0.171	1.156 [0.661–2.022]	0.612
Tertiary	51 (38.1)	83 (61.9)	0.693 [0.417–1.151]	0.157	0.793 [0.411–1.528]	0.488
**Local Government Area**						
Fagge	112 (56.0)	88 (44.0)	0.097 [0.058–0.164]	<0.00I^*^	0.068 [0.033–0.143]	<0.001^*^
Gwale	88 (35.2)	162 (64.8)	0.228 [0.136–0.380]	<0.00I^*^	0.180 [0.091–0.355]	<0.001^*^
Tarauni	22 (11.0)	178 (89.0)	**Ref**	–	–	–
**Drug abuse**						
Never Abused Drugs	98 (33.8)	192 (66.2)	**Ref**	–	–	–
Ever Abused Drugs	124 (34.4)	236 (65.6)	0.971 [0.701–1.346]	0.862	0.804 [0.471–1.373]	0.424
**Drugs injection**						
Never Injected Drugs	154 (33.9)	300 (66.1)	**Ref**	–	–	–
Ever Injected Drugs	68 (34.7)	128 (65.3)	0.966 [0.679–1.375]	0.849	1.069 [0.625–1.829]	0.807
**Age of first sexual debut**						
Below 13 years	49 (27.7)	128 (72.3)	**Ref**	–	–	–
14 – 17 years	87 (32.3)	182 (67.7)	0.801 [0.528-1.215]	0.296	0.879 [0.494–1.561]	0.659
18 years and above	72 (40.2)	107 (59.8)	0.569 [0.365–0.887]	0.013^*^	0.746 [0.394–1.410]	0.366
**Reason for first sexual intercourse**						
Having fun/love	127 (35.9)	227 (64.1)	**Ref**	–	–	–
Peer pressure	15 (24.2)	47 (75.8)	1.753 [0.943–3.260]	0.076	1.975 [0.948–4.114]	0.069
Rape/Forced	6 (35.3)	11 (64.7)	1.026 [0.371–2.839]	0.961	0.569 [0.187–1.732]	0.321
To obtain money	48 (27.9)	124 (72.1)	1.445 [0.971–2.151]	0.070	0.780 [0.459–1.325]	0.359
Marriage	2 (100.0)	–	**–**	–	–	–
**Sexual partners during the last three months**						
One	44 (38.6)	70 (61.4)	**Ref**	–	–	–
More than one	127 (28.5)	318 (71.5)	1.574 [1.024–2.418]	0.038^*^	1.445 [0.794–2.629]	0.228
**Vaginal sex in the last three months**						
Not Have Vaginal Sex	7 (58.3)	5 (41.7)	**Ref**	–	–	–
Have Vaginal Sex	164 (30.0)	383 (70.0)	3.270 [1.023–10.452]	0.046^*^	5.182 [1.085–24.738]	0.039^*^
**Anal sex in the last three months**						
Not Have Anal Sex	159 (30.2)	368 (69.8)	**Ref**	–	–	–
Have Anal Sex	12 (37.5)	20 (62.5)	0.720 [0.344–1.508]	0.384	0.768 [0.281–2.101]	0.607
**Oral sex in the last three months**						
Not Have Oral Sex	151 (30.6)	343 (69.4)	**Ref**	–	–	–
Have Oral Sex	20 (30.8)	45 (69.2)	0.991 [0.566–1.735]	0.973	1.117 [0.582–2.144]	0.739
**Transactional sex engaged in**						
No transactional sex	32 (47.8)	35 (52.2)	**Ref**	–	–	–
Paid for sex	50 (28.4)	126 (71.6)	2.304 [1.289–4.118]	0.005^*^	1.051 [0.507–2.181]	0.893
Paid & received	15 (21.7)	54 (78.3)	3.291 [1.560–6.943]	0.002^*^	1.221 [0.491–3.039]	0.668
Received gift for sex	74 (30.0)	173 (70.0)	2.137 [1.232–3.710]	0.007^*^	0.729 [0.344–1.547]	0.411
**Condom use in the last three months**						
Always	85 (33.5)	169 (66.5)	**Ref**	–	–	–
Irregularly	86 (28.2)	219 (71.8)	1.281 [0.893–1.837]	0.179	1.169 [0.680–2.011	0.572
**Condom use during the last sexual encounter**						
Not Used Condom	65 (30.4)	149 (69.6)	**Ref**	–	–	–
Used Condom	106 (30.8)	238 (69.2)	0.979 [0.676–1.419]	0.913	1.446 [0.810–2.582]	0.212

* **Significant at p < 0.05; COR- Crude odd ratio; AOR – Adjusted Odd ratio.**

Sexual behaviour factors show that FSWs with more than one sexual partner in the last three months are significantly more willing to take up services (COR: 1.574, P = 0.038), though not significant in the adjusted model. Vaginal sex in the last three months is significantly associated with higher willingness (COR: 3.270, P = 0.046; AOR: 5.182, P = 0.039). Those engaged in transactional sex, especially paid for sex (COR: 2.304, P = 0.005), paid and received a gift (COR: 3.291, P = 0.002; AOR: 1.221), and received a gift for sex (COR: 2.137, P = 0.007), are more willing, though only the latter remains significant in the adjusted model. Condom use, both during the last three months and the last sexual encounter, does not show a significant impact on willingness. However, the pattern of transactional sex engagements highlights significant factors in both crude and adjusted models, suggesting that economic factors play a crucial role in the willingness of FSWs to take up HIV preventive services.

### Types of HIV services the FSWs are willing to take

**Fig 3:**
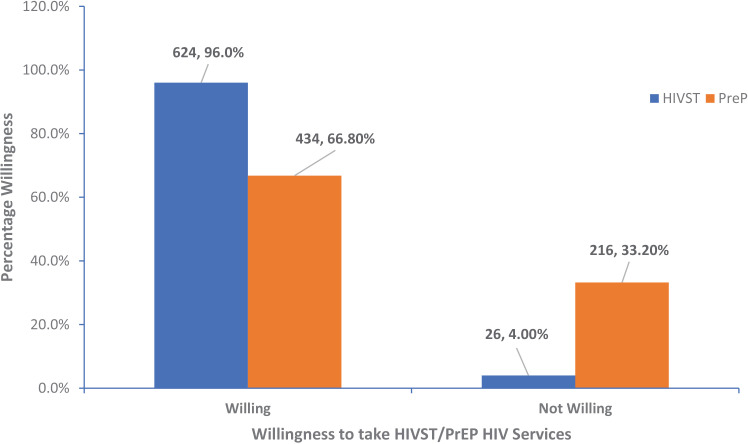
Forms of HIV Services Willingness to Take among FSWs.

Fig. 3 represents the form of HIV services that FSWs are willing to take; 624 (96.0%) are willing to take HIV Self-Test (HIVST) services, while 434 (66.8%) are also indicating willingness to take HIV Pre-exposure Prophylaxis (PrEP) services.

### Reasons for taking HIV Services by FSW

**Fig 4:**
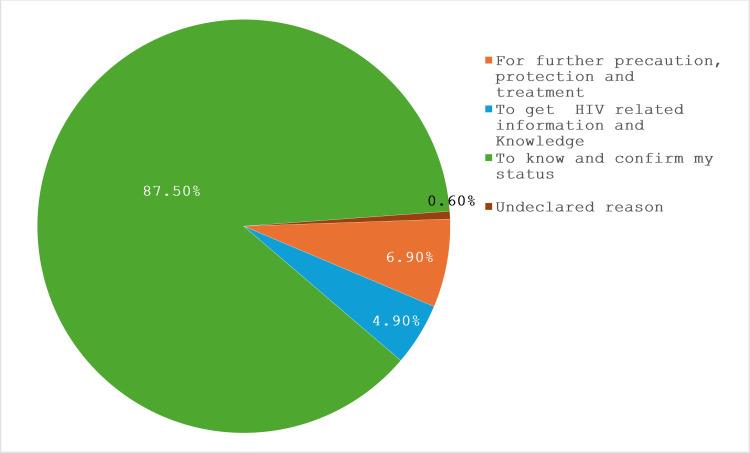
Reasons for taking HIV services.

Fig. 4 shows the reasons reported by the FSWs for taking HIV preventive services. Most of them (87.5%) utilised HIV services to know and confirm their HIV status. On the other hand, 6.9% reported that their reason for taking HIV services was for additional precaution, protection, and treatment, and 4.9% sought HIV services primarily to gain information and knowledge about HIV.

### Obstacles to taking HIV services among FSW

**Fig 5:**
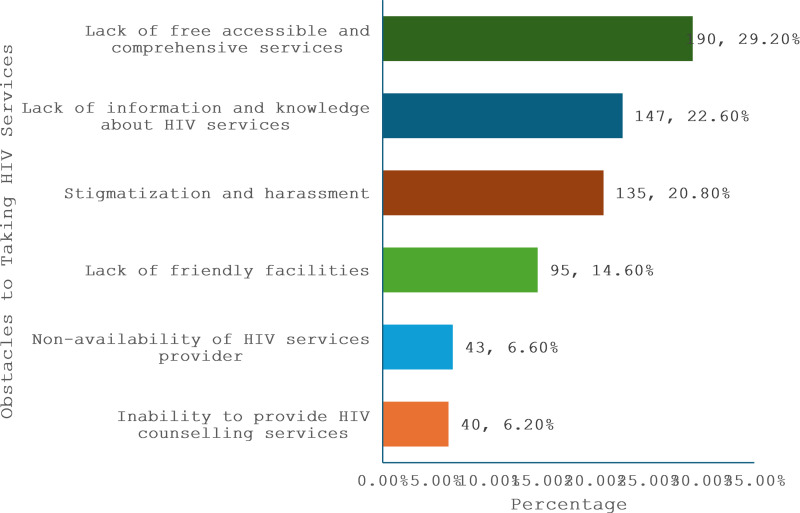
Obstacles to Taking HIV Services.

Fig. 5 illustrates the obstacles faced by FSWs in receiving HIV services. Most FSWs are faced with Unfriendly facilities and stigma related harassment (35.4%) and lack of free accessible and comprehensive services (29.2%). Other obstacles faced includes lack of information and knowledge about HIV services (22.6%), Non availability of HIV services providers (6.6%) and Inability to access HIV counselling services (6.2%).

## Discussion

The findings of this study show that most of the FSWs were 25–34 years old, and one in every five of the population was uneducated. More than half were drug abusers, and 30.2% inject drugs. It was also found that a significant proportion of the FSWs had their first sexual experience before age 13. Fun or affection, peer pressure and financial needs were the major reasons identified for first sexual debut among the respondents, while 2.6% were raped. The majority of the respondents were sexually active in the past three months, with most of them having multiple partners. Oral, anal and vaginal sex were the forms of sexual intercourse prevalent among the FSW, and they were reported receiving gifts and paid for sex. In the past three months, they two fifth were reported using condoms always, and more than half used condoms during their most recent sexual encounter.

### Prevalence of HIV among FSWs in Kano State

The study on the prevalence of HIV and associated factors among FSWs in Kano State reveals an overall HIV prevalence of 8.5%. This overall prevalence observed in this study is in agreement with the study of Bowring *et al*. (8.5%) [20] in Mozambique with low prevalence among FSWs, but in contrast to the findings of Cowan *et al*.[21] who found high HIV prevalence among FSWs in Zimbabwe (57.5%; 42.8–79.2 site minimum and maximum), with significant gaps in the treatment cascade.

### Willingness to take HIV services among FSW

Examining factors associated with the willingness to take HIV preventive services, the study identifies significant determinants. Age shows no significant impact, although FSWs aged 35–45 and above 45 exhibit lower willingness. This contrasts with the findings of Bowring *et al*.[22] where younger FSWs often showed a higher willingness to engage in HIV preventive measures in Cameroon.

Education level similarly does not significantly influence willingness, but FSWs with primary education show a higher willingness. Notably, the Local Government Area (LGA) is a significant factor; FSWs in Fagge and Gwale are significantly less willing to take up services, as indicated by lower crude and adjusted odds ratios. This geographic disparity highlights potential localised barriers to service uptake that warrant further investigation and targeted interventions. This aligns with findings from multi-country studies of Lafort *et al*.[23] which reported the importance of location-specific interventions and that HIV services must be adaptable to different contexts and needs. Behavioural factors also play a crucial role in such a way that the age of first sexual debut impacts willingness, with those debuting at 18 years or older being significantly less willing in crude analysis, although this does not hold in the adjusted model. Peer pressure as a reason for first sexual intercourse indicates a higher, though not significant, willingness to engage with services. Sexual behaviour analysis reveals that FSWs with multiple sexual partners in the last three months show a higher willingness to take up services, aligning with the increased risk perception. Regular engagement in vaginal sex significantly correlates with higher willingness in both crude and adjusted models, suggesting that those perceiving higher risk are more inclined to seek preventive measures. Behavioural factors such as multiple sexual partners and economic motivations (transactional sex) were significant determinants of willingness to engage in HIV services, as observed in Cameroon and other regions by Bowring *et al*.[22] Economic factors are notably influential. Engagement in transactional sex, particularly when paid for sex or receiving gifts for sex, correlates with a higher willingness to take up services. This pattern underscores the critical role of economic motivations in health service engagement among FSWs. This finding has equally shown that significant individuals among the FSWs are willing to take HIV Self-Tests as well as PrEP services amidst the host of other HIV preventive services. The study of Beckham *et al*. supports this.[24] who reported high acceptability of self-testing in both the Dominican Republic and Tanzania and declared that implementing self-testing programs could enhance HIV testing uptake among FSWs. Also, Emmanuel *et al*.[25] findings observed that community support was identified as a facilitator for high PrEP access by key populations in Nigeria.

### Reasons for and against taking HIV services among FSWs

The major reasons identified for accessing HIV services are the desire to know and confirm HIV status (87.5%), followed by the need for additional precaution, protection, and treatment (6.9%), and the pursuit of information and knowledge about HIV (4.9%). These findings emphasise the importance of targeted communication strategies that address specific motivators and barriers to improve service uptake among FSWs in Kano State. However, this finding is consistent with the studies of Milovanovic *et al*.[26] found targeted communication strategies as essential for effective interventions. Also, Lafort *et al*.[27] explored where FSWs seek health care in their study and found out that many FSWs in India, Kenya, Mozambique, and South Africa prefer non-governmental organisations for health services and considered perceived quality and confidentiality as key motivators to accessing HIV services.

Barriers to accessing HIV services are prominently identified. The primary reasons for not accessing services include the lack of free, accessible, and comprehensive services (29.2%), stigmatisation and harassment (20.8%), and a lack of information and knowledge about available services (22.6%). These barriers highlight the need for policy interventions to improve service accessibility, reduce stigma, and enhance information dissemination. These are consistent with findings in South Africa and other regions by Schwartz *et al*.[28] and Emmanuel *et al*.[25] which concluded that FSWs experienced multiple barriers to HIV services uptake, including stigma and lack of support.

### Studt limitation

This study relied on self-reported data for sensitive behaviours, such as drug use and condom use, introducing potential social desirability and recall bias. While anonymity and confidentiality were assured to encourage honesty, these biases may have influenced the accuracy of the findings. The exclusion of other sexually transmitted infections (STIs) and health issues limited the study's scope, leaving a gap in understanding broader health challenges among FSWs. The use of snowball sampling introduced selection bias and limited generalizability, as the sample may overrepresent certain subgroups. Similarly, self-selection bias could have skewed the findings, as FSWs may have been more health-conscious or had better access to services than non-participants. To enhance representativeness and validity, future studies should consider random sampling and targeted outreach to harder-to-reach populations. Finally, stigma and discrimination may have affected participation and honest reporting, potentially impacting the findings. Efforts to mitigate this included discreet recruitment and community engagement, but further research should explore strategies to overcome these barriers and address the limitations identified.

## Conclusion

This study underscores how socio-economic challenges, health inequities, and stigma shape HIV prevalence and service uptake among FSWs in Kano State. Addressing systemic barriers, including limited access to free healthcare and stigma, requires holistic interventions such as community-led programs, economic empowerment, and tailored health services. These strategies can reduce HIV prevalence and improve outcomes, serving as a model for similar contexts. Future research should assess the long-term impact of interventions, explore socio-cultural and behavioural factors, and evaluate innovative approaches like mobile and digital health tools to enhance service accessibility and sustainability. Also Studies should also assess innovative approaches, such as mobile clinics and digital health tools, to enhance service accessibility and uptake.

## Supporting information

S1 Data FSW Kano Data. (XLSX)
